# An operative barrier system for skull base and mastoid surgery: creating a safe operative theatre in the era of COVID-19

**DOI:** 10.1186/s40463-020-00471-0

**Published:** 2020-10-06

**Authors:** Justin Cottrell, Justin Lui, Trung Le, Joseph Chen

**Affiliations:** 1 Sunnybrook Health Science Centre, University of Toronto, Toronto, Ontario Canada; 2 Department of Otolaryngology – Head & Neck Surgery, University of Toronto, St. George Campus, David Naylor Building, 6 Queen’s Park Crescent West, Suite 120, Toronto, ON M5S 3H2 Canada

**Keywords:** COVID-19, AGMP, Neurotology, Mastoidectomy, Healthcare safety, Barrier system

## Abstract

Within Neurotology, special draping systems have been devised for mastoid surgery recognizing that drilling of middle ear mucosa is an aerosol generating medical procedure (AGMP) which can place surgical teams at risk of COVID-19 infection. We provide a thorough description of a barrier system utilized in our practice, along with work completed by our group to better quantify its effectiveness. Utilization of a barrier system can provide near complete bone dust and droplet containment within the surgical field and prevent contamination of other healthcare workers. As this is an early system, further adaptations and national collaborations are required to ultimately arrive at a system that seamlessly integrates into the surgical suite. While these barrier systems are new, they are timely as we face a pandemic, and can play a crucial role in safely resuming surgery.

## Introduction

A major challenge in the provision of care during the COVID-19 pandemic is to secure personal protective equipment (PPE) while working to curb the spread of this novel coronavirus. As hospitals prepare to resume surgical activities, the concern for false negative screening tests combined with shortages of N95 masks have highlighted the need for additional safeguards when performing potentially aerosol generating medical procedures (AGMPs) [1]. In the case of otologic surgeries, the middle ear has demonstrated concomitant viral colonization from the nasopharynx [2–4] raising concerns for dispersion due to the need for prolonged drilling, irrigation and suctioning. The aerosolized bone dust and large amounts of droplets may infect healthcare providers [2–5].

## Use of an operative barrier system for AGMPs

Simple barrier draping systems have been described with some published experiences. However, restricted mobility, adaptability, and additional equipment purchases or customizations have limited widespread adoption [6, 7]. We sought to improve upon prior designs by utilizing readily available operative equipment to suspend a barrier drape over the surgical field, to promote superior surgical ergonomics and droplet containment, while preserving operative visibility (Additional file 1). This system is now being utilized for urgent neurotology surgeries in our practice in addition to other protective practices such as pre-operative COVID-19 testing 48 h before surgery when feasible. Individual personal protective equipment for the surgical team includes gown, gloves, and surgical mask, with the addition of face shield when not working under the microscope. The circulating nurse and anesthetist utilize a surgical mask at all times, adding N95 mask, gown, gloves and face shield when intubating and extubating. As we move forward to facilitate elective surgeries, we believe the barrier system outlined will play an important role in the provision of safe surgical management.

## What is the evidence so far?

Current evidence to support the use of barrier systems in AGMPs involve quantification of droplet dispersion in simulated settings. The barrier system proposed has been assessed by our group through the simulation of a mastoidectomy in the operative theatre. In addition to the inclusion of a surgeon and assistant, a manikin was created to simulate a scrub nurse. A polymer-based 3D printed temporal bone was bolstered into a simulated patients’ Styrofoam head for drilling. Surgical irrigation containing fluorescein dye (1 mg/mL) was used to visualize droplet contamination.

Two mastoidectomies were performed under standard draping protocol, one with and one without the barrier system. Following 15 min of drilling with a 6 mm cutting burr at speeds up to 70,000 RPM, the surgical site was examined under UV light. The droplet dispersion characteristics were compared between the two protocols.

**Fig. 1:**
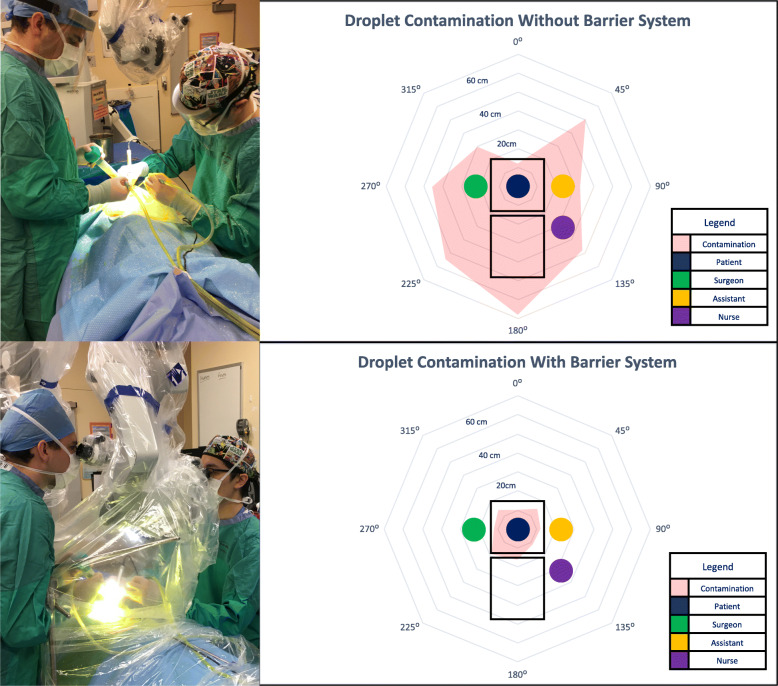
Droplet Contamination with and Without Barrier System

The barrier system provided near complete bone dust and droplet containment within the surgical field (Fig. 1). Without it, dispersion was found up to 68 in. away from the ear, with frank contamination of the facemask, shield, exposed skin, and gown seen on the operative team (see Temporal Bone Drilling Video and Additional file 2). No contamination was found on any part of the team members except for the gloved hands of the surgeon and assistant using the barrier system. The participants noted no visualization interference and minimal ergonomic limitations.

In addition to simulated experience, the authors have found the system easy to incorporate in the urgent neurotology cases performed to date.

## What are the harms?

Droplet containment appears to be superior with a barrier system for mastoidectomy, however care must still be taken when passing instruments and removing the drape following a procedure. Incorporating a Mayo stand within the enclosure with frequently used instruments can reduce the amount of barrier breaches required to pass instruments. Slow removal of the drape after cessation of drilling and tying the redundant drape material to the microscope can help prevent aerosolization that may result from manipulation.

While it may be reasonably inferred that droplet protection will also help minimize aerosolization, this has not been directly studied, and would not have been accurately captured in our described experience. Continuing to follow evolving guidance documents, literature on transmission risk, along with infectious disease best practices such as hand hygiene and proper doffing of surgical masks and gowns should still be advised [8, 9].

## What can we expect in the future?

This novel design concept could potentially be applied to high risk AGMP procedures in other anatomical regions in the head & neck system and would be suitable for microscopic and endoscopic platforms with some modifications. As this is an early system, further adaptations and national collaborations are required to ultimately arrive at a system that seamlessly integrates into the surgical suite. Working to develop more effective and efficient means of pre-operative COVID-19 testing, as well as other perioperative prevention strategies will continue to play an important role of an overall preventative strategy. As we struggle to deal with the backlog of semi-urgent and elective procedures from COVID-19 restrictions, prioritizing research advances in barrier devices can assist in the safe return of patients, health professionals, and learners to the operating rooms in Canada.


**Additional file 3: Video 2.** Drape Placement Tutorial. Additional visualization of drape assembly.

## Supplementary information

**Additional file 1.** Additional information describing equipment, the barrier assembly, and additional contamination results without the barrier system.

**Additional file 2: Video 1.** Temporal Bone Drilling, Contamination Visualized with Fluorescein. Additional visualization of drilling contamination utilizing UV light and fluorescein. (MOV 7506 kb)

## Data Availability

The datasets used and/or analysed during the current study are available from the corresponding author on reasonable request.

## Abbreviations

PPE: Personal protective equipment
AGMP: Aerosol generating medical procedures

## References

[1] Li W, Huang J, Guo X, Zhao J, Mandell S. Anesthesia management and perioperative infection control in patients with the novel coronavirus. J Cardiothorac Vasc Anesth. 2020:1–6. 10.1053/j.jvca.2020.03.035. 10.1053/j.jvca.2020.03.035 PMC7146651 32279934

[2] Heikkenen T, Thint M, Chonmaitree T (1999). Prevalence of various respiratory viruses in the middle ear during acute otitis media. N Engl J Med.

[3] Frazier K, Hooper J, Mostafa H, Stewart M. SARS-CoV-2 virus isolated from the mastoid and middle ear: implications for COVID-19 precautions during ear surgery. JAMA Otolaryngol Head Neck Surg. 2020;23. 10.1001/jamaoto.2020.1922. 10.1001/jamaoto.2020.1922 PMC7378866 32701126

[4] Thamboo A, Lea J, Sommer D (2020). Clinical evidence based review and recommendations of aerosol generating medical procedures in otolaryngology – head and neck surgery during the COVID-19 pandemic. J Otolaryngol Head Neck Surg..

[5] Givi B, Schiff BA, Chinn SB, et al. Safety recommendations for evaluation and surgery of the head and neck during the COVID-19 pandemic. JAMA Otolaryngol Head Neck Surg. 2020;31. 10.1001/jamaoto.2020.0780. 10.1001/jamaoto.2020.0780 32232423

[6] Carron JD, Buck LS, Harbarger CF, Eby TL. A simple technique for droplet control during mastoid surgery. JAMA Otolaryngol Head Neck Surg. 2020;28. 10.1001/jamaoto.2020.1064. 10.1001/jamaoto.2020.1064 PMC7189332 32343347

[7] Chen J, Workman A, Chari D, et al. Demonstration and mitigation of aerosol and particle dispersion during Mastoidectomy relevant to the COVID-19 era. Otol Neurotol. 2020;8. 10.1097/MAO.0000000000002765. 10.1097/MAO.0000000000002765 PMC7497894 32925848

[8] Lammers M, Lea J, Westerberg B (2020). Guidance for Otolaryngology Health Care Workers Performing Aerosol Generating Medical Procedures During the COVID-19 Pandemic. J Otolaryngol Head Neck Surg.

[9] Mick P, Murphy R (2020). Aerosol-generating otolaryngology procedures and the need for enhanced PPE during the COVID-19 pandemic: a literature review. J Otolaryngol Head Neck Surg.

