# Supplementary material for: An operative barrier system for skull base and mastoid surgery: creating a safe operative theatre in the era of COVID-19
Source: J Otolaryngol Head Neck Surg. 2020 Oct 6;49:71. doi: 10.1186/s40463-020-00471-0 (PMC7537966; doi:10.1186/s40463-020-00471-0)

Supplementary Appendix

Table of Contents

A. Equipment
B. Barrier Assembly
C. Additional Contamination Results Without Barrier

A. Equipment

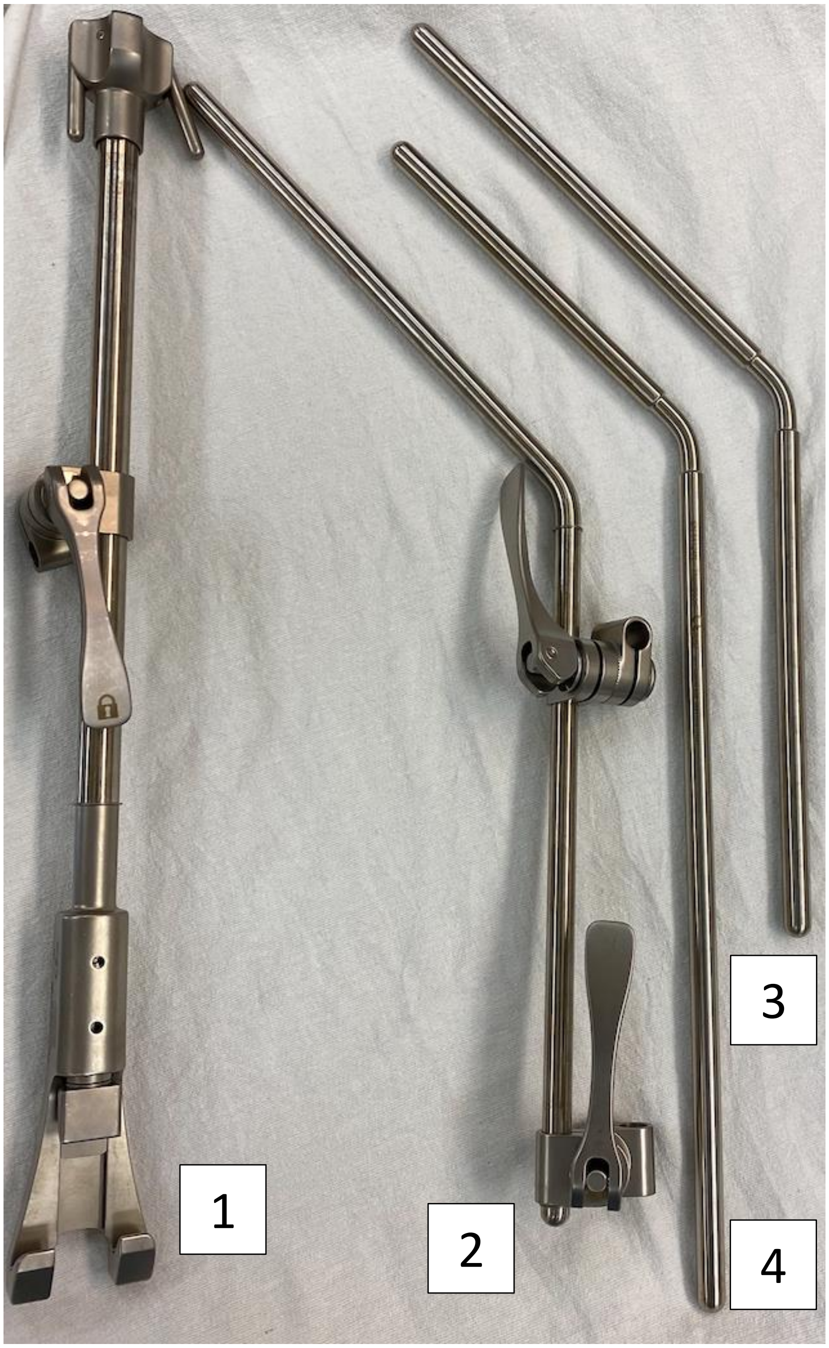

1. Thompson 43902AC - Elite III Rail Clamp 18” with cam joint
2. Thompson 43900BC - Crossbar with 2 Cam III Joints 24” (11”x 13”)
3. Thompson 44120 - Angles Lateral Arm 20” (10” x 10”)
4. Thompson 44124N - Angled Lateral Arm 24” (8” x 16”)
5. Genesys C-Arm / Mobile X-Ray Drape (41” x 74”) (NOT SHOWN)

B. Barrier Assembly

Step 1: Prep and Drape the Patient in Sterile Fashion

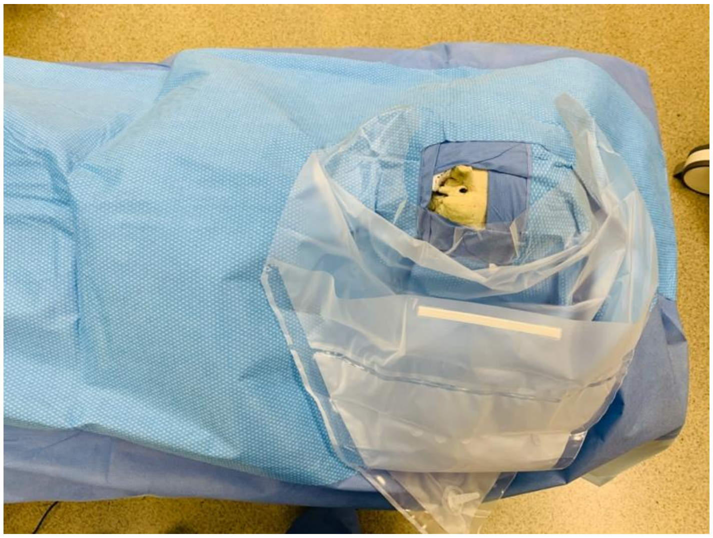

Step 2: Assemble the Barrier Framework Over Sterile Field

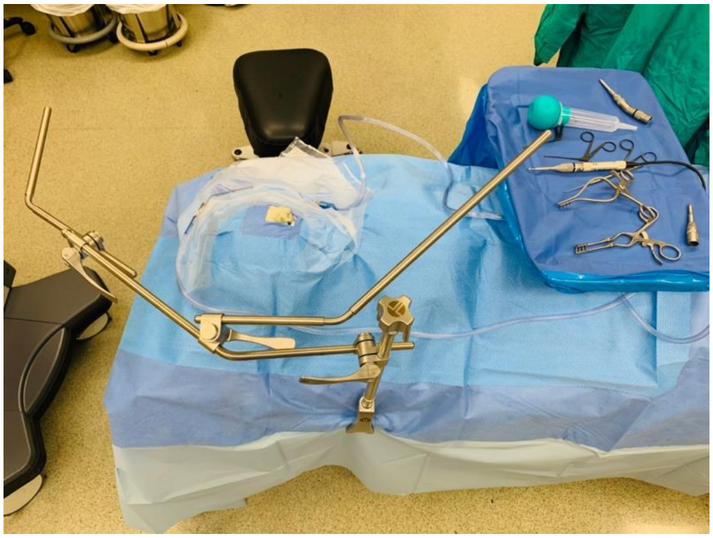

Step 3: Following Standard Microscope Draping Procedure, Cut Hole in C-Arm X-Ray Drape (Barrier Drape) and Wrap Around the Microscope from Below Lens. Secure Barrier Drape to Microscope Drape with Elastic Bands (or Sterile Tape). Release Barrier Drape from Microscope to Cover Surgical Field and Secure with Clamps to Bed (See Drape Placement Tutorial Video)

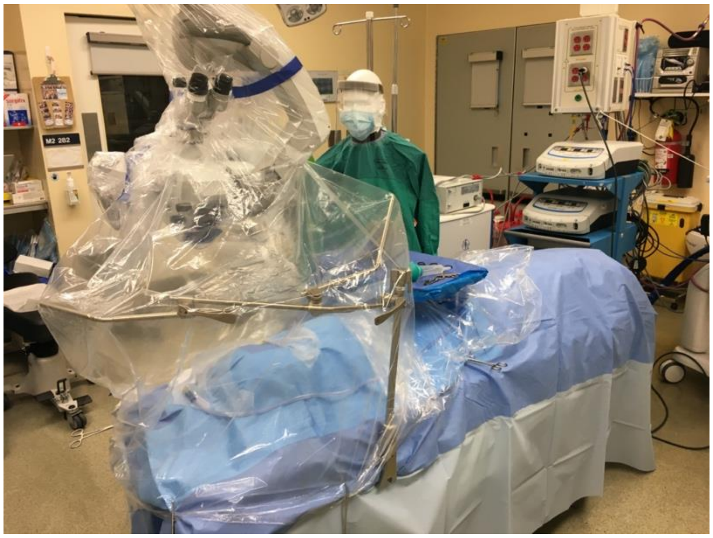

Step 4: Surgeon and Assistant Evaluates Optimal Placement For Hands in the Drape. Using Scissors, Cut Small Holes for Hand Access (option: Include Mayo Tray Within Enclosure)

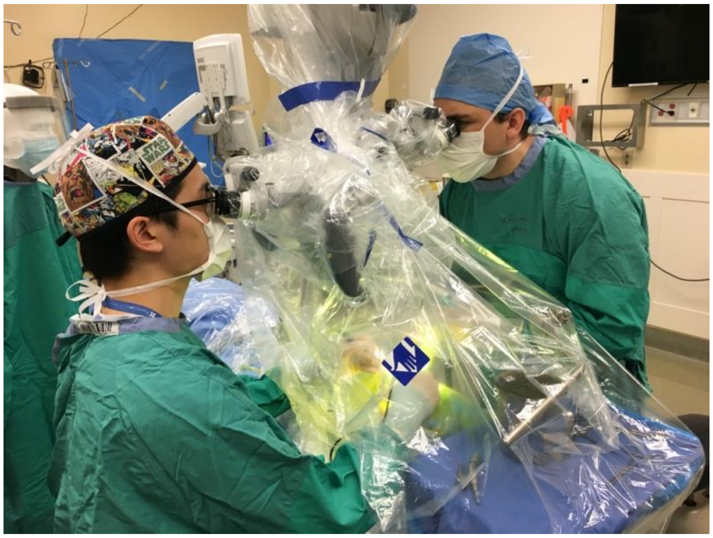

C. Additional Contamination Results Without Barrier

1. Post-Operative Surgical Field

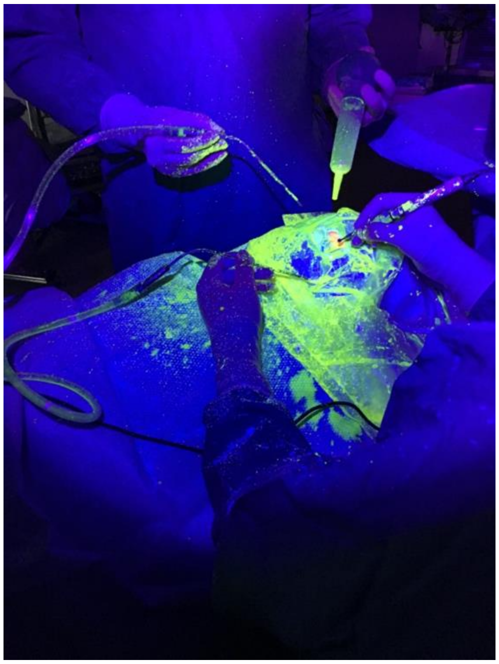

2. Surgeon Gown Contamination

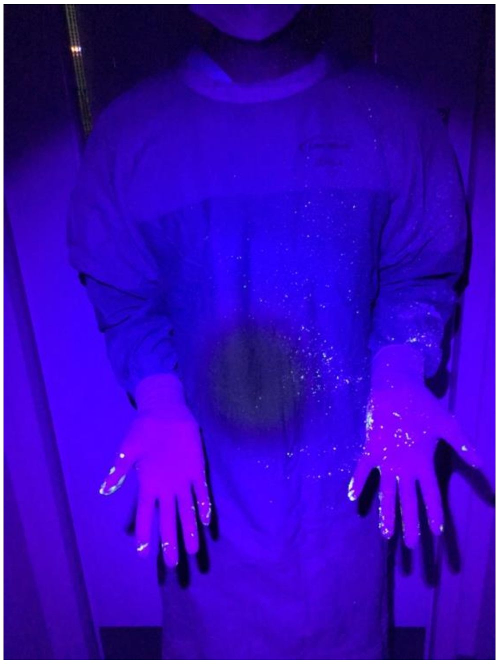

(Wet fluorescein stained yellow, dry fluorescein stained red)

3. Surgeon Faceshield Contamination

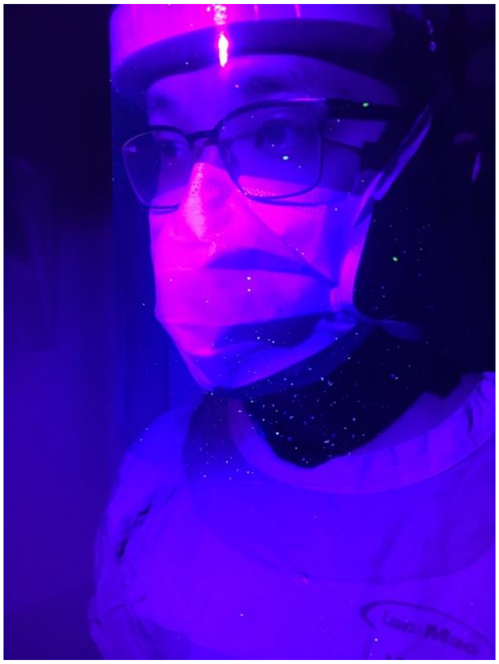

4. Assistant Neck and Mask Contamination Behind Faceshield

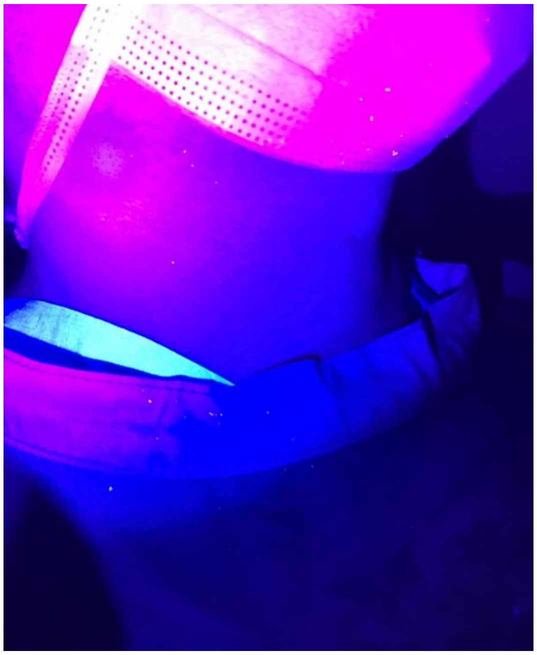

Supplement: Supplementary file 2 — Additional file 1. Additional information describing equipment, the barrier assembly, and additional contamination results without the barrier system. [file 40463_2020_471_MOESM1_ESM.pdf]
